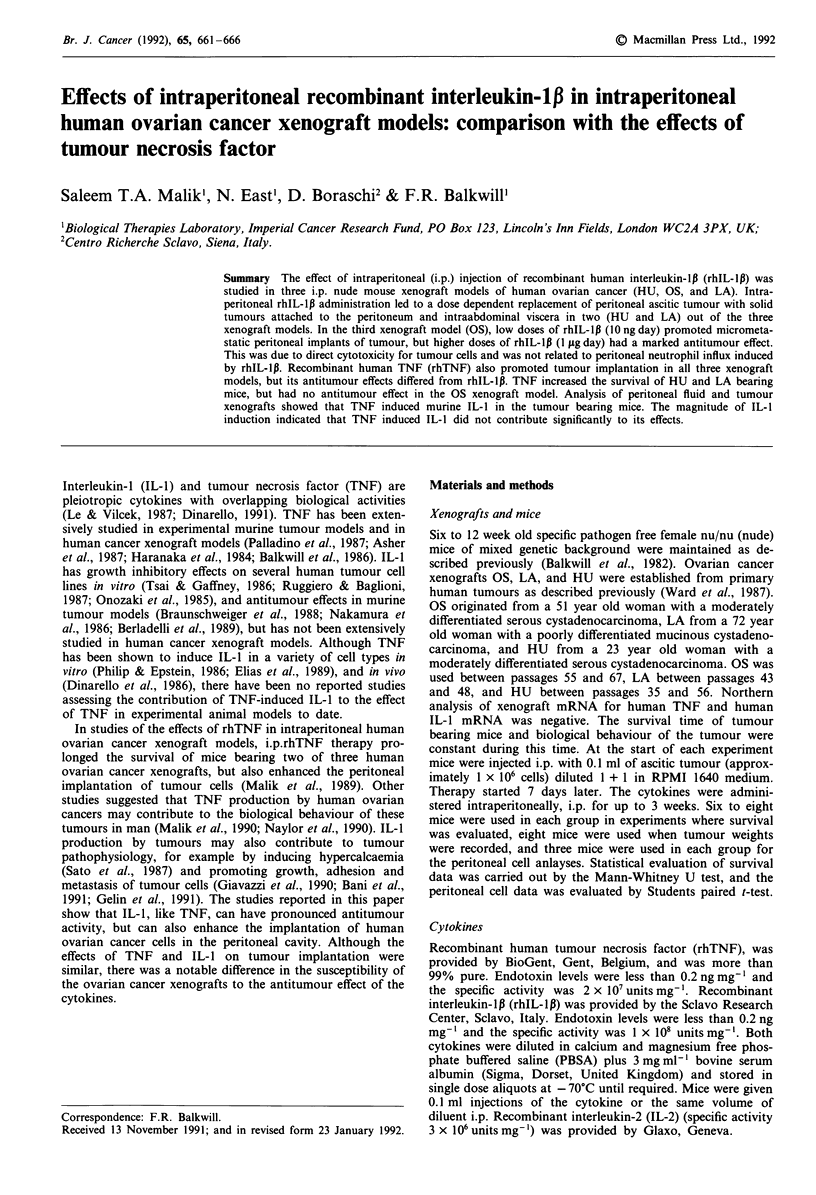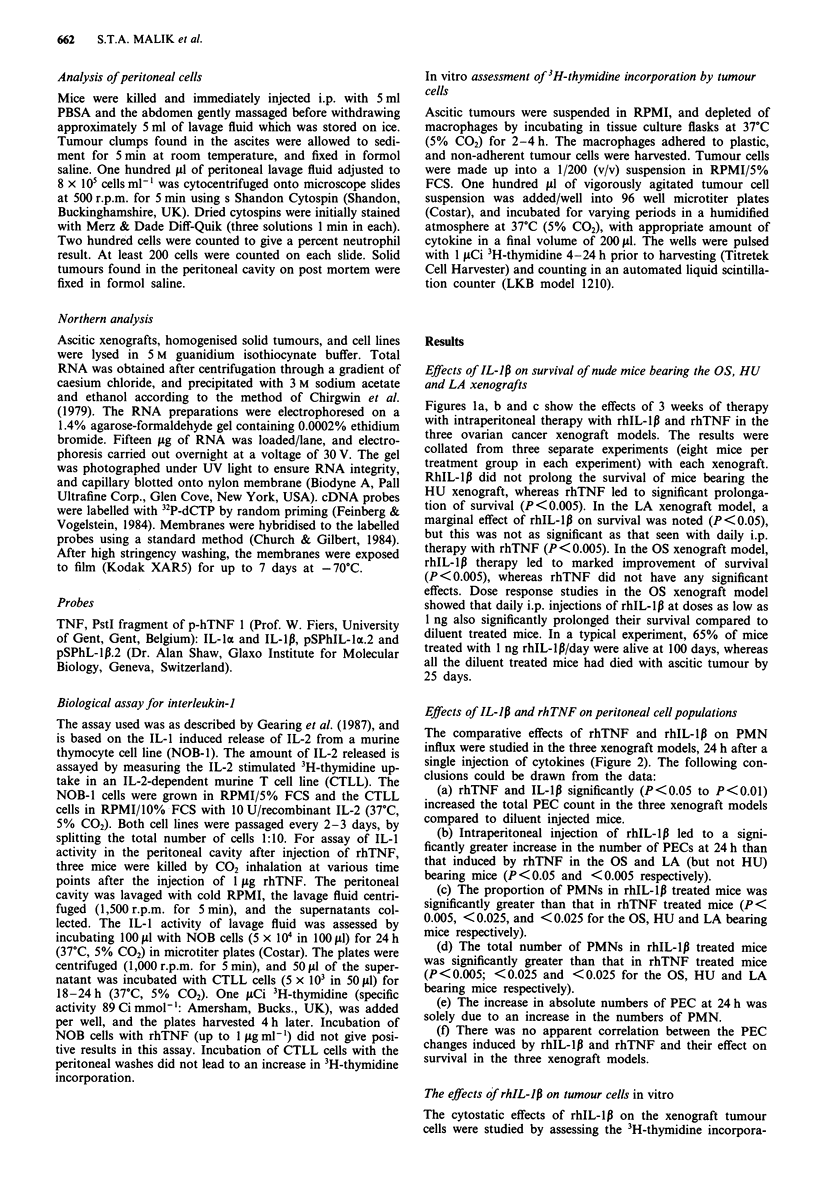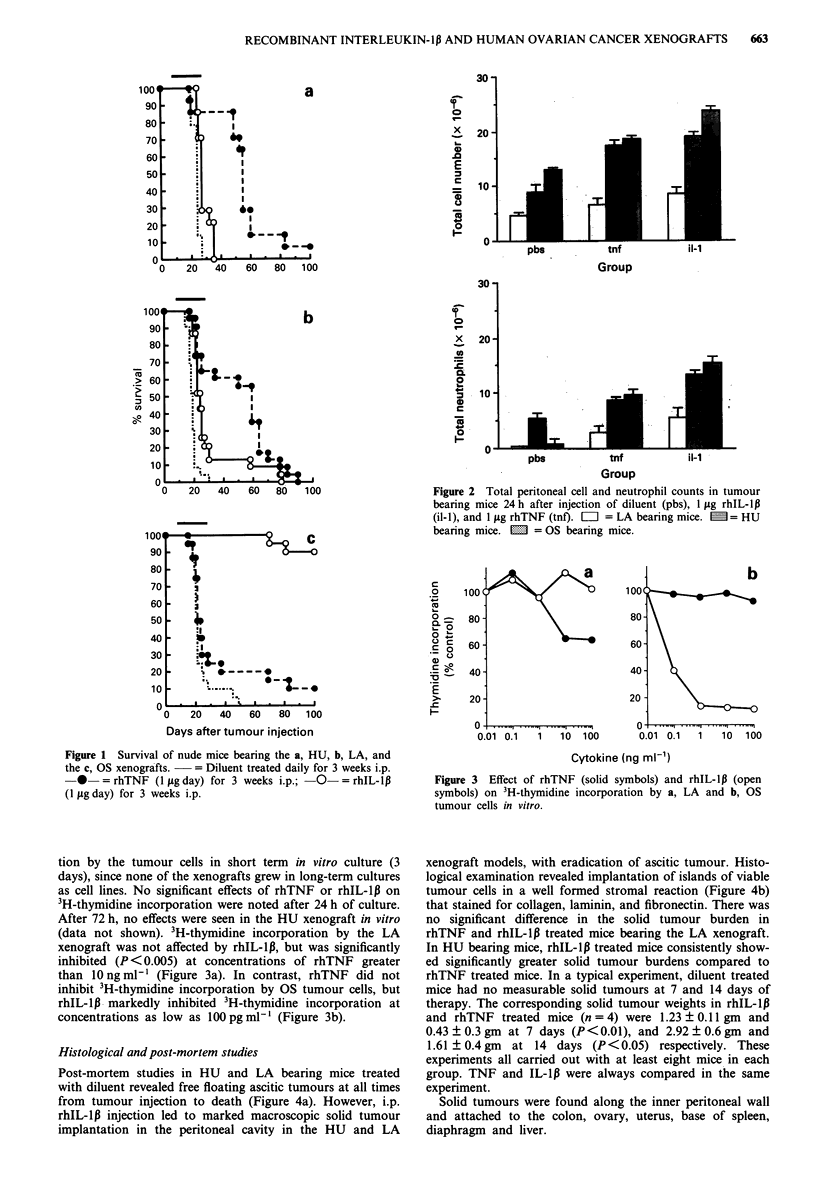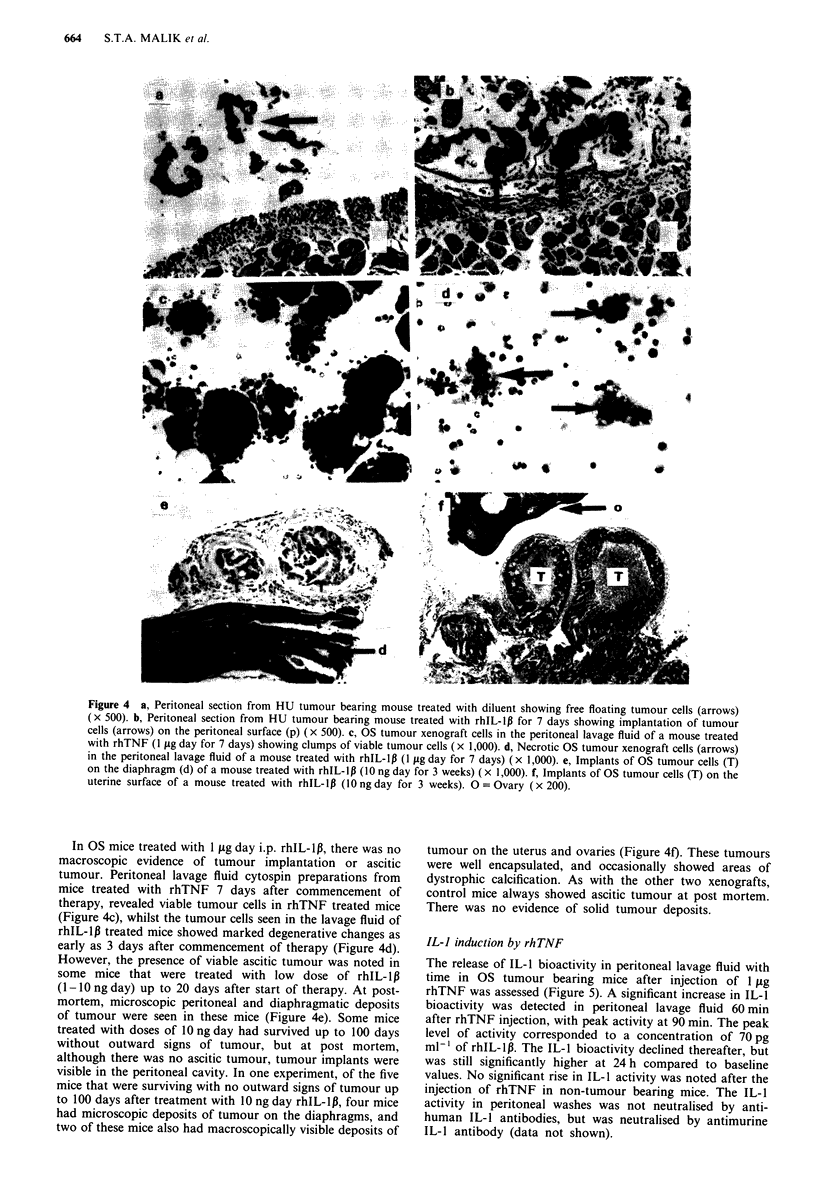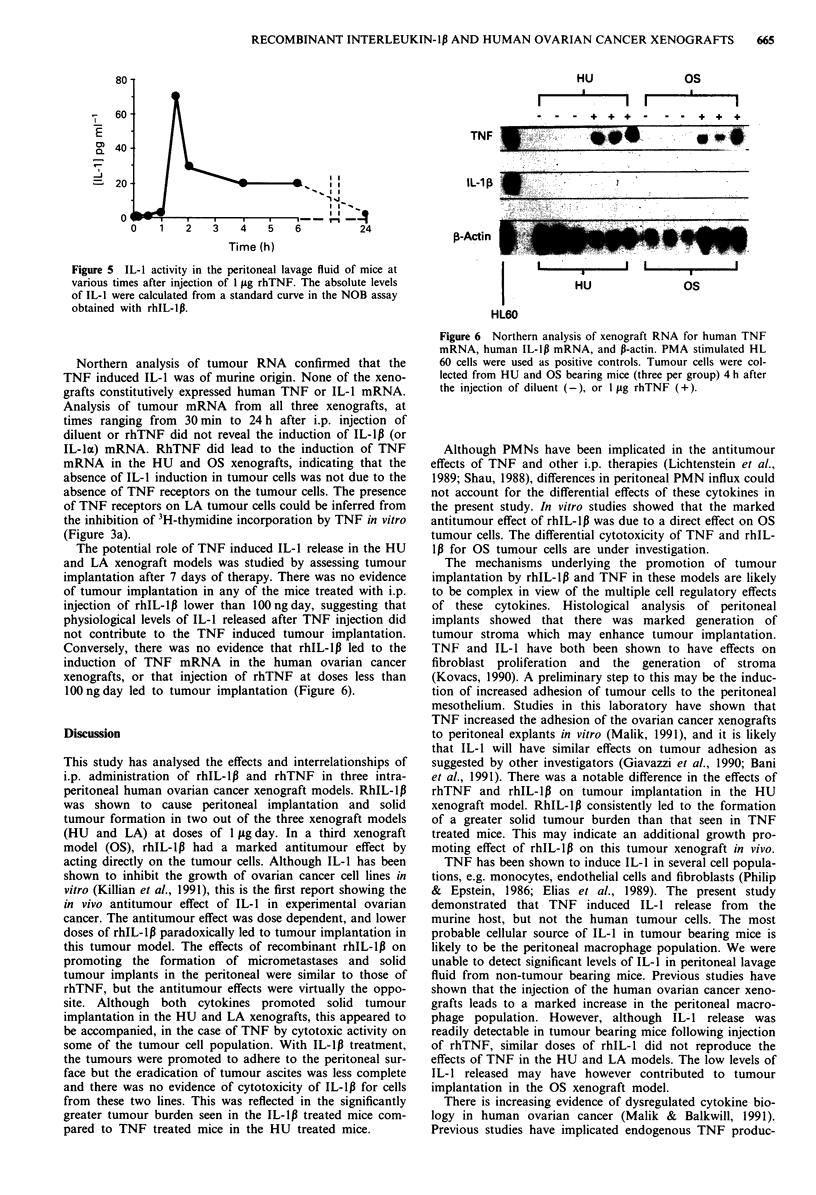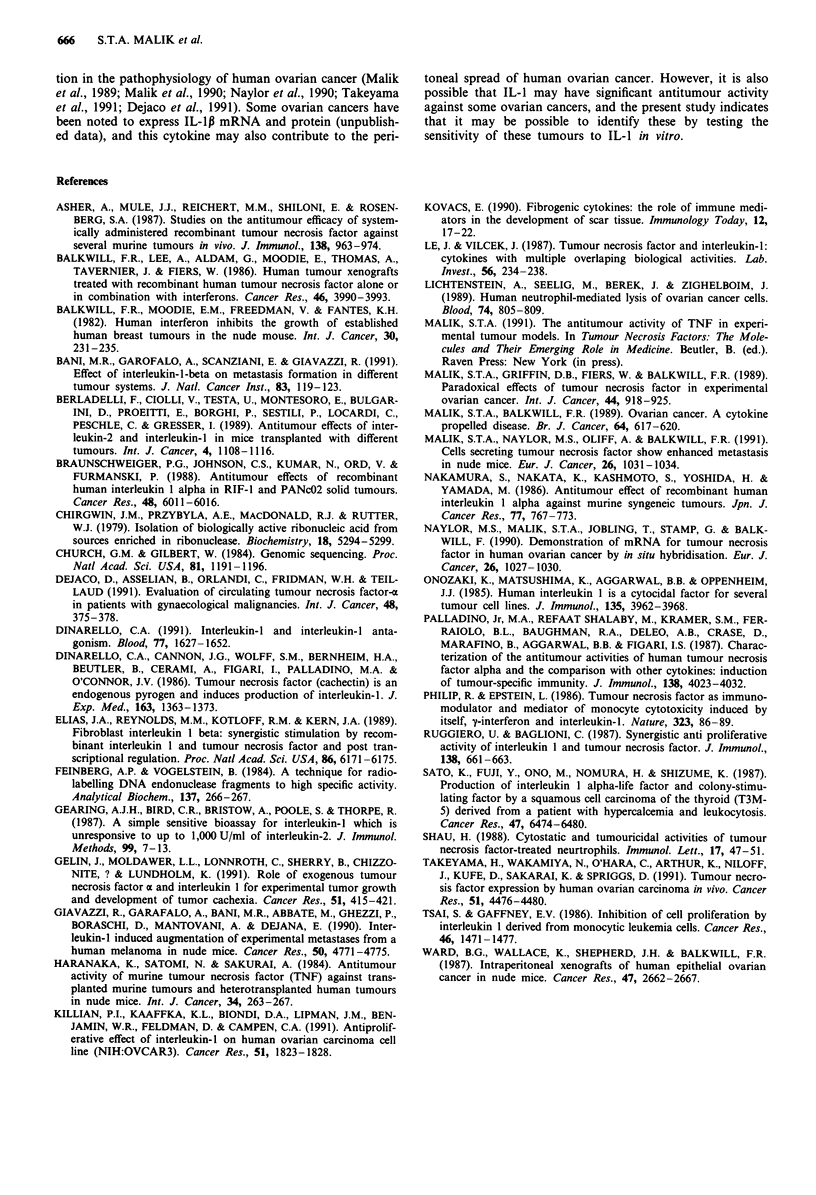# Effects of intraperitoneal recombinant interleukin-1 beta in intraperitoneal human ovarian cancer xenograft models: comparison with the effects of tumour necrosis factor.

**DOI:** 10.1038/bjc.1992.141

**Published:** 1992-05

**Authors:** S. T. Malik, N. East, D. Boraschi, F. R. Balkwill

**Affiliations:** Biological Therapies Laboratory, Imperial Cancer Research Fund, London, UK.

## Abstract

**Images:**


Br. J. Cancer (1992), 65, 661-666 © Macmillan Press Ltd., 1992

### Effects of intraperitoneal recombinant interleukin-1β in intraperitoneal human ovarian cancer xenograft models: comparison with the effects of tumour necrosis factor

Saleem T.A. Malik[1], N. East[1], D. Boraschi[2] & F.R. Balkwill[1]

[1]*Biological Therapies Laboratory, Imperial Cancer Research Fund, PO Box 123, Lincoln's Inn Fields, London WC2A 3PX, UK;* [2]*Centro Richerche Sclavo, Siena, Italy.*

**Summary** The effect of intraperitoneal (i.p.) injection of recombinant human interleukin-1β (rhIL-1β) was studied in three i.p. nude mouse xenograft models of human ovarian cancer (HU, OS, and LA). Intraperitoneal rhIL-1β administration led to a dose dependent replacement of peritoneal ascitic tumour with solid tumours attached to the peritoneum and intraabdominal viscera in two (HU and LA) out of the three xenograft models. In the third xenograft model (OS), low doses of rhIL-1β (10 ng day) promoted micrometastatic peritoneal implants of tumour, but higher doses of rhIL-1β (1 μg day) had a marked antitumour effect. This was due to direct cytotoxicity for tumour cells and was not related to peritoneal neutrophil influx induced by rhIL-1β. Recombinant human TNF (rhTNF) also promoted tumour implantation in all three xenograft models, but its antitumour effects differed from rhIL-1β. TNF increased the survival of HU and LA bearing mice, but had no antitumour effect in the OS xenograft model. Analysis of peritoneal fluid and tumour xenografts showed that TNF induced murine IL-1 in the tumour bearing mice. The magnitude of IL-1 induction indicated that TNF induced IL-1 did not contribute significantly to its effects.

Interleukin-1 (IL-1) and tumour necrosis factor (TNF) are pleiotropic cytokines with overlapping biological activities (Le & Vilcek, 1987; Dinarello, 1991). TNF has been extensively studied in experimental murine tumour models and in human cancer xenograft models (Palladino *et al.*, 1987; Asher *et al.*, 1987; Haranaka *et al.*, 1984; Balkwill *et al.*, 1986). IL-1 has growth inhibitory effects on several human tumour cell lines *in vitro* (Tsai & Gaffney, 1986; Ruggiero & Baglioni, 1987; Onozaki *et al.*, 1985), and antitumour effects in murine tumour models (Braunschweiger *et al.*, 1988; Nakamura *et al.*, 1986; Berladelli *et al.*, 1989), but has not been extensively studied in human cancer xenograft models. Although TNF has been shown to induce IL-1 in a variety of cell types *in vitro* (Philip & Epstein, 1986; Elias *et al.*, 1989), and *in vivo* (Dinarello *et al.*, 1986), there have been no reported studies assessing the contribution of TNF-induced IL-1 to the effect of TNF in experimental animal models to date.

In studies of the effects of rhTNF in intraperitoneal human ovarian cancer xenograft models, i.p.rhTNF therapy prolonged the survival of mice bearing two of three human ovarian cancer xenografts, but also enhanced the peritoneal implantation of tumour cells (Malik *et al.*, 1989). Other studies suggested that TNF production by human ovarian cancers may contribute to the biological behaviour of these tumours in man (Malik *et al.*, 1990; Naylor *et al.*, 1990). IL-1 production by tumours may also contribute to tumour pathophysiology, for example by inducing hypercalcaemia (Sato *et al.*, 1987) and promoting growth, adhesion and metastasis of tumour cells (Giavazzi *et al.*, 1990; Bani *et al.*, 1991; Gelin *et al.*, 1991). The studies reported in this paper show that IL-1, like TNF, can have pronounced antitumour activity, but can also enhance the implantation of human ovarian cancer cells in the peritoneal cavity. Although the effects of TNF and IL-1 on tumour implantation were similar, there was a notable difference in the susceptibility of the ovarian cancer xenografts to the antitumour effect of the cytokines.

Correspondence: F.R. Balkwill.
Received 13 November 1991; and in revised form 23 January 1992.

### Materials and methods

### *Xenografts and mice*

Six to 12 week old specific pathogen free female nu/nu (nude) mice of mixed genetic background were maintained as described previously (Balkwill *et al.*, 1982). Ovarian cancer xenografts OS, LA, and HU were established from primary human tumours as described previously (Ward *et al.*, 1987). OS originated from a 51 year old woman with a moderately differentiated serous cystadenocarcinoma, LA from a 72 year old woman with a poorly differentiated mucinous cystadenocarcinoma, and HU from a 23 year old woman with a moderately differentiated serous cystadenocarcinoma. OS was used between passages 55 and 67, LA between passages 43 and 48, and HU between passages 35 and 56. Northern analysis of xenograft mRNA for human TNF and human IL-1 mRNA was negative. The survival time of tumour bearing mice and biological behaviour of the tumour were constant during this time. At the start of each experiment mice were injected i.p. with 0.1 ml of ascitic tumour (approximately $1 \times 10^6$ cells) diluted 1 + 1 in RPMI 1640 medium. Therapy started 7 days later. The cytokines were administered intraperitoneally, i.p. for up to 3 weeks. Six to eight mice were used in each group in experiments where survival was evaluated, eight mice were used when tumour weights were recorded, and three mice were used in each group for the peritoneal cell anlayses. Statistical evaluation of survival data was carried out by the Mann-Whitney U test, and the peritoneal cell data was evaluated by Students paired *t*-test.

### *Cytokines*

Recombinant human tumour necrosis factor (rhTNF), was provided by BioGent, Gent, Belgium, and was more than 99% pure. Endotoxin levels were less than 0.2 ng $mg^{-1}$ and the specific activity was $2 \times 10^7$ units $mg^{-1}$. Recombinant interleukin-1β (rhIL-1β) was provided by the Sclavo Research Center, Sclavo, Italy. Endotoxin levels were less than 0.2 ng $mg^{-1}$ and the specific activity was $1 \times 10^8$ units $mg^{-1}$. Both cytokines were diluted in calcium and magnesium free phosphate buffered saline (PBSA) plus 3 mg $ml^{-1}$ bovine serum albumin (Sigma, Dorset, United Kingdom) and stored in single dose aliquots at $-70°C$ until required. Mice were given 0.1 ml injections of the cytokine or the same volume of diluent i.p. Recombinant interleukin-2 (IL-2) (specific activity $3 \times 10^6$ units $mg^{-1}$) was provided by Glaxo, Geneva.

*Analysis of peritoneal cells*

Mice were killed and immediately injected i.p. with 5 ml PBSA and the abdomen gently massaged before withdrawing approximately 5 ml of lavage fluid which was stored on ice. Tumour clumps found in the ascites were allowed to sediment for 5 min at room temperature, and fixed in formol saline. One hundred μl of peritoneal lavage fluid adjusted to $8 \times 10^5$ cells $ml^{-1}$ was cytocentrifuged onto microscope slides at 500 r.p.m. for 5 min using s Shandon Cytospin (Shandon, Buckinghamshire, UK). Dried cytospins were initially stained with Merz & Dade Diff-Quik (three solutions 1 min in each). Two hundred cells were counted to give a percent neutrophil result. At least 200 cells were counted on each slide. Solid tumours found in the peritoneal cavity on post mortem were fixed in formol saline.

*Northern analysis*

Ascitic xenografts, homogenised solid tumours, and cell lines were lysed in 5 M guanidium isothiocynate buffer. Total RNA was obtained after centrifugation through a gradient of caesium chloride, and precipitated with 3 M sodium acetate and ethanol according to the method of Chirgwin *et al.* (1979). The RNA preparations were electrophoresed on a 1.4% agarose-formaldehyde gel containing 0.0002% ethidium bromide. Fifteen μg of RNA was loaded/lane, and electrophoresis carried out overnight at a voltage of 30 V. The gel was photographed under UV light to ensure RNA integrity, and capillary blotted onto nylon membrane (Biodyne A, Pall Ultrafine Corp., Glen Cove, New York, USA). cDNA probes were labelled with $^{32}$P-dCTP by random priming (Feinberg & Vogelstein, 1984). Membranes were hybridised to the labelled probes using a standard method (Church & Gilbert, 1984). After high stringency washing, the membranes were exposed to film (Kodak XAR5) for up to 7 days at −70°C.

*Probes*

TNF, PstI fragment of p-hTNF 1 (Prof. W. Fiers, University of Gent, Gent, Belgium): IL-1α and IL-1β, pSPhIL-1α.2 and pSPhL-1β.2 (Dr. Alan Shaw, Glaxo Institute for Molecular Biology, Geneva, Switzerland).

*Biological assay for interleukin-1*

The assay used was as described by Gearing *et al.* (1987), and is based on the IL-1 induced release of IL-2 from a murine thymocyte cell line (NOB-1). The amount of IL-2 released is assayed by measuring the IL-2 stimulated $^3$H-thymidine uptake in an IL-2-dependent murine T cell line (CTLL). The NOB-1 cells were grown in RPMI/5% FCS and the CTLL cells in RPMI/10% FCS with 10 U/recombinant IL-2 (37°C, 5% $CO_2$). Both cell lines were passaged every 2–3 days, by splitting the total number of cells 1:10. For assay of IL-1 activity in the peritoneal cavity after injection of rhTNF, three mice were killed by $CO_2$ inhalation at various time points after the injection of 1 μg rhTNF. The peritoneal cavity was lavaged with cold RPMI, the lavage fluid centrifuged (1,500 r.p.m. for 5 min), and the supernatants collected. The IL-1 activity of lavage fluid was assessed by incubating 100 μl with NOB cells ($5 \times 10^4$ in 100 μl) for 24 h (37°C, 5% $CO_2$) in microtiter plates (Costar). The plates were centrifuged (1,000 r.p.m. for 5 min), and 50 μl of the supernatant was incubated with CTLL cells ($5 \times 10^3$ in 50 μl) for 18–24 h (37°C, 5% $CO_2$). One μCi $^3$H-thymidine (specific activity 89 Ci $mmol^{-1}$: Amersham, Bucks., UK), was added per well, and the plates harvested 4 h later. Incubation of NOB cells with rhTNF (up to 1 μg $ml^{-1}$) did not give positive results in this assay. Incubation of CTLL cells with the peritoneal washes did not lead to an increase in $^3$H-thymidine incorporation.

In vitro *assessment of $^3$H-thymidine incorporation by tumour cells*

Ascitic tumours were suspended in RPMI, and depleted of macrophages by incubating in tissue culture flasks at 37°C (5% $CO_2$) for 2–4 h. The macrophages adhered to plastic, and non-adherent tumour cells were harvested. Tumour cells were made up into a 1/200 (v/v) suspension in RPMI/5% FCS. One hundred μl of vigorously agitated tumour cell suspension was added/well into 96 well microtiter plates (Costar), and incubated for varying periods in a humidified atmosphere at 37°C (5% $CO_2$), with appropriate amount of cytokine in a final volume of 200 μl. The wells were pulsed with 1 μCi $^3$H-thymidine 4–24 h prior to harvesting (Titretek Cell Harvester) and counting in an automated liquid scintillation counter (LKB model 1210).

### Results

*Effects of IL-1β on survival of nude mice bearing the OS, HU and LA xenografts*

Figures 1a, b and c show the effects of 3 weeks of therapy with intraperitoneal therapy with rhIL-1β and rhTNF in the three ovarian cancer xenograft models. The results were collated from three separate experiments (eight mice per treatment group in each experiment) with each xenograft. RhIL-1β did not prolong the survival of mice bearing the HU xenograft, whereas rhTNF led to significant prolongation of survival ($P < 0.005$). In the LA xenograft model, a marginal effect of rhIL-1β on survival was noted ($P < 0.05$), but this was not as significant as that seen with daily i.p. therapy with rhTNF ($P < 0.005$). In the OS xenograft model, rhIL-1β therapy led to marked improvement of survival ($P < 0.005$), whereas rhTNF did not have any significant effects. Dose response studies in the OS xenograft model showed that daily i.p. injections of rhIL-1β at doses as low as 1 ng also significantly prolonged their survival compared to diluent treated mice. In a typical experiment, 65% of mice treated with 1 ng rhIL-1β/day were alive at 100 days, whereas all the diluent treated mice had died with ascitic tumour by 25 days.

*Effects of IL-1β and rhTNF on peritoneal cell populations*

The comparative effects of rhTNF and rhIL-1β on PMN influx were studied in the three xenograft models, 24 h after a single injection of cytokines (Figure 2). The following conclusions could be drawn from the data:

(a) rhTNF and IL-1β significantly ($P < 0.05$ to $P < 0.01$) increased the total PEC count in the three xenograft models compared to diluent injected mice.

(b) Intraperitoneal injection of rhIL-1β led to a significantly greater increase in the number of PECs at 24 h than that induced by rhTNF in the OS and LA (but not HU) bearing mice ($P < 0.05$ and $< 0.005$ respectively).

(c) The proportion of PMNs in rhIL-1β treated mice was significantly greater than that in rhTNF treated mice ($P <$ 0.005, $< 0.025$, and $< 0.025$ for the OS, HU and LA bearing mice respectively).

(d) The total number of PMNs in rhIL-1β treated mice was significantly greater than that in rhTNF treated mice ($P < 0.005$; $< 0.025$ and $< 0.025$ for the OS, HU and LA bearing mice respectively).

(e) The increase in absolute numbers of PEC at 24 h was solely due to an increase in the numbers of PMN.

(f) There was no apparent correlation between the PEC changes induced by rhIL-1β and rhTNF and their effect on survival in the three xenograft models.

*The effects of rhIL-1β on tumour cells* in vitro

The cytostatic effects of rhIL-1β on the xenograft tumour cells were studied by assessing the $^3$H-thymidine incorpora-

**Figure 1** Survival of nude mice bearing the **a**, HU, **b**, LA, and the **c**, OS xenografts. —— = Diluent treated daily for 3 weeks i.p. —●— = rhTNF (1 μg day) for 3 weeks i.p.; —O— = rhIL-1β (1 μg day) for 3 weeks i.p.

**Figure 2** Total peritoneal cell and neutrophil counts in tumour bearing mice 24 h after injection of diluent (pbs), 1 μg rhIL-1β (il-1), and 1 μg rhTNF (tnf). ☐ = LA bearing mice. ▤ = HU bearing mice. ▩ = OS bearing mice.

**Figure 3** Effect of rhTNF (solid symbols) and rhIL-1β (open symbols) on $^3$H-thymidine incorporation by **a**, LA and **b**, OS tumour cells *in vitro*.

tion by the tumour cells in short term *in vitro* culture (3 days), since none of the xenografts grew in long-term cultures as cell lines. No significant effects of rhTNF or rhIL-1β on $^3$H-thymidine incorporation were noted after 24 h of culture. After 72 h, no effects were seen in the HU xenograft *in vitro* (data not shown). $^3$H-thymidine incorporation by the LA xenograft was not affected by rhIL-1β, but was significantly inhibited ($P < 0.005$) at concentrations of rhTNF greater than 10 ng ml$^{-1}$ (Figure 3a). In contrast, rhTNF did not inhibit $^3$H-thymidine incorporation by OS tumour cells, but rhIL-1β markedly inhibited $^3$H-thymidine incorporation at concentrations as low as 100 pg ml$^{-1}$ (Figure 3b).

### *Histological and post-mortem studies*

Post-mortem studies in HU and LA bearing mice treated with diluent revealed free floating ascitic tumours at all times from tumour injection to death (Figure 4a). However, i.p. rhIL-1β injection led to marked macroscopic solid tumour implantation in the peritoneal cavity in the HU and LA xenograft models, with eradication of ascitic tumour. Histological examination revealed implantation of islands of viable tumour cells in a well formed stromal reaction (Figure 4b) that stained for collagen, laminin, and fibronectin. There was no significant difference in the solid tumour burden in rhTNF and rhIL-1β treated mice bearing the LA xenograft. In HU bearing mice, rhIL-1β treated mice consistently showed significantly greater solid tumour burdens compared to rhTNF treated mice. In a typical experiment, diluent treated mice had no measurable solid tumours at 7 and 14 days of therapy. The corresponding solid tumour weights in rhIL-1β and rhTNF treated mice ($n = 4$) were $1.23 \pm 0.11$ gm and $0.43 \pm 0.3$ gm at 7 days ($P < 0.01$), and $2.92 \pm 0.6$ gm and $1.61 \pm 0.4$ gm at 14 days ($P < 0.05$) respectively. These experiments all carried out with at least eight mice in each group. TNF and IL-1β were always compared in the same experiment.

Solid tumours were found along the inner peritoneal wall and attached to the colon, ovary, uterus, base of spleen, diaphragm and liver.

**Figure 4** **a**, Peritoneal section from HU tumour bearing mouse treated with diluent showing free floating tumour cells (arrows) (× 500). **b**, Peritoneal section from HU tumour bearing mouse treated with rhIL-1β for 7 days showing implantation of tumour cells (arrows) on the peritoneal surface (p) (× 500). **c**, OS tumour xenograft cells in the peritoneal lavage fluid of a mouse treated with rhTNF (1 μg day for 7 days) showing clumps of viable tumour cells (× 1,000). **d**, Necrotic OS tumour xenograft cells (arrows) in the peritoneal lavage fluid of a mouse treated with rhIL-1β (1 μg day for 7 days) (× 1,000). **e**, Implants of OS tumour cells (T) on the diaphragm (d) of a mouse treated with rhIL-1β (10 ng day for 3 weeks) (× 1,000). **f**, Implants of OS tumour cells (T) on the uterine surface of a mouse treated with rhIL-1β (10 ng day for 3 weeks). O = Ovary (× 200).

In OS mice treated with 1 μg day i.p. rhIL-1β, there was no macroscopic evidence of tumour implantation or ascitic tumour. Peritoneal lavage fluid cytospin preparations from mice treated with rhTNF 7 days after commencement of therapy, revealed viable tumour cells in rhTNF treated mice (Figure 4c), whilst the tumour cells seen in the lavage fluid of rhIL-1β treated mice showed marked degenerative changes as early as 3 days after commencement of therapy (Figure 4d). However, the presence of viable ascitic tumour was noted in some mice that were treated with low dose of rhIL-1β (1–10 ng day) up to 20 days after start of therapy. At post-mortem, microscopic peritoneal and diaphragmatic deposits of tumour were seen in these mice (Figure 4e). Some mice treated with doses of 10 ng day had survived up to 100 days without outward signs of tumour, but at post mortem, although there was no ascitic tumour, tumour implants were visible in the peritoneal cavity. In one experiment, of the five mice that were surviving with no outward signs of tumour up to 100 days after treatment with 10 ng day rhIL-1β, four mice had microscopic deposits of tumour on the diaphragms, and two of these mice also had macroscopically visible deposits of tumour on the uterus and ovaries (Figure 4f). These tumours were well encapsulated, and occasionally showed areas of dystrophic calcification. As with the other two xenografts, control mice always showed ascitic tumour at post mortem. There was no evidence of solid tumour deposits.

### *IL-1 induction by rhTNF*

The release of IL-1 bioactivity in peritoneal lavage fluid with time in OS tumour bearing mice after injection of 1 μg rhTNF was assessed (Figure 5). A significant increase in IL-1 bioactivity was detected in peritoneal lavage fluid 60 min after rhTNF injection, with peak activity at 90 min. The peak level of activity corresponded to a concentration of 70 pg $ml^{-1}$ of rhIL-1β. The IL-1 bioactivity declined thereafter, but was still significantly higher at 24 h compared to baseline values. No significant rise in IL-1 activity was noted after the injection of rhTNF in non-tumour bearing mice. The IL-1 activity in peritoneal washes was not neutralised by anti-human IL-1 antibodies, but was neutralised by antimurine IL-1 antibody (data not shown).

**Figure 5** IL-1 activity in the peritoneal lavage fluid of mice at various times after injection of 1 μg rhTNF. The absolute levels of IL-1 were calculated from a standard curve in the NOB assay obtained with rhIL-1β.

**Figure 6** Northern analysis of xenograft RNA for human TNF mRNA, human IL-1β mRNA, and β-actin. PMA stimulated HL 60 cells were used as positive controls. Tumour cells were collected from HU and OS bearing mice (three per group) 4 h after the injection of diluent (−), or 1 μg rhTNF (+).

Northern analysis of tumour RNA confirmed that the TNF induced IL-1 was of murine origin. None of the xenografts constitutively expressed human TNF or IL-1 mRNA. Analysis of tumour mRNA from all three xenografts, at times ranging from 30 min to 24 h after i.p. injection of diluent or rhTNF did not reveal the induction of IL-1β (or IL-1α) mRNA. RhTNF did lead to the induction of TNF mRNA in the HU and OS xenografts, indicating that the absence of IL-1 induction in tumour cells was not due to the absence of TNF receptors on the tumour cells. The presence of TNF receptors on LA tumour cells could be inferred from the inhibition of $^3$H-thymidine incorporation by TNF *in vitro* (Figure 3a).

The potential role of TNF induced IL-1 release in the HU and LA xenograft models was studied by assessing tumour implantation after 7 days of therapy. There was no evidence of tumour implantation in any of the mice treated with i.p. injection of rhIL-1β lower than 100 ng day, suggesting that physiological levels of IL-1 released after TNF injection did not contribute to the TNF induced tumour implantation. Conversely, there was no evidence that rhIL-1β led to the induction of TNF mRNA in the human ovarian cancer xenografts, or that injection of rhTNF at doses less than 100 ng day led to tumour implantation (Figure 6).

### Discussion

This study has analysed the effects and interrelationships of i.p. administration of rhIL-1β and rhTNF in three intraperitoneal human ovarian cancer xenograft models. RhIL-1β was shown to cause peritoneal implantation and solid tumour formation in two out of the three xenograft models (HU and LA) at doses of 1 μg day. In a third xenograft model (OS), rhIL-1β had a marked antitumour effect by acting directly on the tumour cells. Although IL-1 has been shown to inhibit the growth of ovarian cancer cell lines *in vitro* (Killian *et al.*, 1991), this is the first report showing the *in vivo* antitumour effect of IL-1 in experimental ovarian cancer. The antitumour effect was dose dependent, and lower doses of rhIL-1β paradoxically led to tumour implantation in this tumour model. The effects of recombinant rhIL-1β on promoting the formation of micrometastases and solid tumour implants in the peritoneal were similar to those of rhTNF, but the antitumour effects were virtually the opposite. Although both cytokines promoted solid tumour implantation in the HU and LA xenografts, this appeared to be accompanied, in the case of TNF by cytotoxic activity on some of the tumour cell population. With IL-1β treatment, the tumours were promoted to adhere to the peritoneal surface but the eradication of tumour ascites was less complete and there was no evidence of cytotoxicity of IL-1β for cells from these two lines. This was reflected in the significantly greater tumour burden seen in the IL-1β treated mice compared to TNF treated mice in the HU treated mice.

Although PMNs have been implicated in the antitumour effects of TNF and other i.p. therapies (Lichtenstein *et al.*, 1989; Shau, 1988), differences in peritoneal PMN influx could not account for the differential effects of these cytokines in the present study. *In vitro* studies showed that the marked antitumour effect of rhIL-1β was due to a direct effect on OS tumour cells. The differential cytotoxicity of TNF and rhIL-1β for OS tumour cells are under investigation.

The mechanisms underlying the promotion of tumour implantation by rhIL-1β and TNF in these models are likely to be complex in view of the multiple cell regulatory effects of these cytokines. Histological analysis of peritoneal implants showed that there was marked generation of tumour stroma which may enhance tumour implantation. TNF and IL-1 have both been shown to have effects on fibroblast proliferation and the generation of stroma (Kovacs, 1990). A preliminary step to this may be the induction of increased adhesion of tumour cells to the peritoneal mesothelium. Studies in this laboratory have shown that TNF increased the adhesion of the ovarian cancer xenografts to peritoneal explants *in vitro* (Malik, 1991), and it is likely that IL-1 will have similar effects on tumour adhesion as suggested by other investigators (Giavazzi *et al.*, 1990; Bani *et al.*, 1991). There was a notable difference in the effects of rhTNF and rhIL-1β on tumour implantation in the HU xenograft model. RhIL-1β consistently led to the formation of a greater solid tumour burden than that seen in TNF treated mice. This may indicate an additional growth promoting effect of rhIL-1β on this tumour xenograft *in vivo*.

TNF has been shown to induce IL-1 in several cell populations, e.g. monocytes, endothelial cells and fibroblasts (Philip & Epstein, 1986; Elias *et al.*, 1989). The present study demonstrated that TNF induced IL-1 release from the murine host, but not the human tumour cells. The most probable cellular source of IL-1 in tumour bearing mice is likely to be the peritoneal macrophage population. We were unable to detect significant levels of IL-1 in peritoneal lavage fluid from non-tumour bearing mice. Previous studies have shown that the injection of the human ovarian cancer xenografts leads to a marked increase in the peritoneal macrophage population. However, although IL-1 release was readily detectable in tumour bearing mice following injection of rhTNF, similar doses of rhIL-1 did not reproduce the effects of TNF in the HU and LA models. The low levels of IL-1 released may have however contributed to tumour implantation in the OS xenograft model.

There is increasing evidence of dysregulated cytokine biology in human ovarian cancer (Malik & Balkwill, 1991). Previous studies have implicated endogenous TNF production in the pathophysiology of human ovarian cancer (Malik et al., 1989; Malik et al., 1990; Naylor et al., 1990; Takeyama et al., 1991; Dejaco et al., 1991). Some ovarian cancers have been noted to express IL-1β mRNA and protein (unpublished data), and this cytokine may also contribute to the peritoneal spread of human ovarian cancer. However, it is also possible that IL-1 may have significant antitumour activity against some ovarian cancers, and the present study indicates that it may be possible to identify these by testing the sensitivity of these tumours to IL-1 *in vitro*.

### References

ASHER, A., MULE, J.J., REICHERT, M.M., SHILONI, E. & ROSENBERG, S.A. (1987). Studies on the antitumour efficacy of systemically administered recombinant tumour necrosis factor against several murine tumours *in vivo*. *J. Immunol.*, **138**, 963–974.

BALKWILL, F.R., LEE, A., ALDAM, G., MOODIE, E., THOMAS, A., TAVERNIER, J. & FIERS, W. (1986). Human tumour xenografts treated with recombinant human tumour necrosis factor alone or in combination with interferons. *Cancer Res.*, **46**, 3990–3993.

BALKWILL, F.R., MOODIE, E.M., FREEDMAN, V. & FANTES, K.H. (1982). Human interferon inhibits the growth of established human breast tumours in the nude mouse. *Int. J. Cancer*, **30**, 231–235.

BANI, M.R., GAROFALO, A., SCANZIANI, E. & GIAVAZZI, R. (1991). Effect of interleukin-1-beta on metastasis formation in different tumour systems. *J. Natl. Cancer Inst.*, **83**, 119–123.

BERLADELLI, F., CIOLLI, V., TESTA, U., MONTESORO, E., BULGARINI, D., PROEITTI, E., BORGHI, P., SESTILI, P., LOCARDI, C., PESCHLE, C. & GRESSER, I. (1989). Antitumour effects of interleukin-2 and interleukin-1 in mice transplanted with different tumours. *Int. J. Cancer*, **4**, 1108–1116.

BRAUNSCHWEIGER, P.G., JOHNSON, C.S., KUMAR, N., ORD, V. & FURMANSKI, P. (1988). Antitumour effects of recombinant human interleukin 1 alpha in RIF-1 and PANc02 solid tumours. *Cancer Res.*, **48**, 6011–6016.

CHIRGWIN, J.M., PRZYBYLA, A.E., MACDONALD, R.J. & RUTTER, W.J. (1979). Isolation of biologically active ribonucleic acid from sources enriched in ribonuclease. *Biochemistry*, **18**, 5294–5299.

CHURCH, G.M. & GILBERT, W. (1984). Genomic sequencing. *Proc. Natl Acad. Sci. USA*, **81**, 1191–1196.

DEJACO, D., ASSELIAN, B., ORLANDI, C., FRIDMAN, W.H. & TEILLAUD (1991). Evaluation of circulating tumour necrosis factor-α in patients with gynaecological malignancies. *Int. J. Cancer*, **48**, 375–378.

DINARELLO, C.A. (1991). Interleukin-1 and interleukin-1 antagonism. *Blood*, **77**, 1627–1652.

DINARELLO, C.A., CANNON, J.G., WOLFF, S.M., BERNHEIM, H.A., BEUTLER, B., CERAMI, A., FIGARI, I., PALLADINO, M.A. & O'CONNOR, J.V. (1986). Tumour necrosis factor (cachectin) is an endogenous pyrogen and induces production of interleukin-1. *J. Exp. Med.*, **163**, 1363–1373.

ELIAS, J.A., REYNOLDS, M.M., KOTLOFF, R.M. & KERN, J.A. (1989). Fibroblast interleukin 1 beta: synergistic stimulation by recombinant interleukin 1 and tumour necrosis factor and post transcriptional regulation. *Proc. Natl Acad. Sci. USA*, **86**, 6171–6175.

FEINBERG, A.P. & VOGELSTEIN, B. (1984). A technique for radiolabelling DNA endonuclease fragments to high specific activity. *Analytical Biochem.*, **137**, 266–267.

GEARING, A.J.H., BIRD, C.R., BRISTOW, A., POOLE, S. & THORPE, R. (1987). A simple sensitive bioassay for interleukin-1 which is unresponsive to up to 1,000 U/ml of interleukin-2. *J. Immunol. Methods*, **99**, 7–13.

GELIN, J., MOLDAWER, L.L., LONNROTH, C., SHERRY, B., CHIZZONITE, ? & LUNDHOLM, K. (1991). Role of exogenous tumour necrosis factor α and interleukin 1 for experimental tumor growth and development of tumor cachexia. *Cancer Res.*, **51**, 415–421.

GIAVAZZI, R., GARAFALO, A., BANI, M.R., ABBATE, M., GHEZZI, P., BORASCHI, D., MANTOVANI, A. & DEJANA, E. (1990). Interleukin-1 induced augmentation of experimental metastases from a human melanoma in nude mice. *Cancer Res.*, **50**, 4771–4775.

HARANAKA, K., SATOMI, N. & SAKURAI, A. (1984). Antitumour activity of murine tumour necrosis factor (TNF) against transplanted murine tumours and heterotransplanted human tumours in nude mice. *Int. J. Cancer*, **34**, 263–267.

KILLIAN, P.I., KAAFFKA, K.L., BIONDI, D.A., LIPMAN, J.M., BENJAMIN, W.R., FELDMAN, D. & CAMPEN, C.A. (1991). Antiproliferative effect of interleukin-1 on human ovarian carcinoma cell line (NIH:OVCAR3). *Cancer Res.*, **51**, 1823–1828.

KOVACS, E. (1990). Fibrogenic cytokines: the role of immune mediators in the development of scar tissue. *Immunology Today*, **12**, 17–22.

LE, J. & VILCEK, J. (1987). Tumour necrosis factor and interleukin-1: cytokines with multiple overlaping biological activities. *Lab. Invest.*, **56**, 234–238.

LICHTENSTEIN, A., SEELIG, M., BEREK, J. & ZIGHELBOIM, J. (1989). Human neutrophil-mediated lysis of ovarian cancer cells. *Blood*, **74**, 805–809.

MALIK, S.T.A. (1991). The antitumour activity of TNF in experimental tumour models. In *Tumour Necrosis Factors: The Molecules and Their Emerging Role in Medicine*. Beutler, B. (ed.). Raven Press: New York (in press).

MALIK, S.T.A., GRIFFIN, D.B., FIERS, W. & BALKWILL, F.R. (1989). Paradoxical effects of tumour necrosis factor in experimental ovarian cancer. *Int. J. Cancer*, **44**, 918–925.

MALIK, S.T.A., BALKWILL, F.R. (1989). Ovarian cancer. A cytokine propelled disease. *Br. J. Cancer*, **64**, 617–620.

MALIK, S.T.A., NAYLOR, M.S., OLIFF, A. & BALKWILL, F.R. (1991). Cells secreting tumour necrosis factor show enhanced metastasis in nude mice. *Eur. J. Cancer*, **26**, 1031–1034.

NAKAMURA, S., NAKATA, K., KASHMOTO, S., YOSHIDA, H. & YAMADA, M. (1986). Antitumour effect of recombinant human interleukin 1 alpha against murine syngeneic tumours. *Jpn. J. Cancer Res.*, **77**, 767–773.

NAYLOR, M.S., MALIK, S.T.A., JOBLING, T., STAMP, G. & BALKWILL, F. (1990). Demonstration of mRNA for tumour necrosis factor in human ovarian cancer by *in situ* hybridisation. *Eur. J. Cancer*, **26**, 1027–1030.

ONOZAKI, K., MATSUSHIMA, K., AGGARWAL, B.B. & OPPENHEIM, J.J. (1985). Human interleukin 1 is a cytocidal factor for several tumour cell lines. *J. Immunol.*, **135**, 3962–3968.

PALLADINO, Jr, M.A., REFAAT SHALABY, M., KRAMER, S.M., FERRAIOLO, B.L., BAUGHMAN, R.A., DELEO, A.B., CRASE, D., MARAFINO, B., AGGARWAL, B.B. & FIGARI, I.S. (1987). Characterization of the antitumour activities of human tumour necrosis factor alpha and the comparison with other cytokines: induction of tumour-specific immunity. *J. Immunol.*, **138**, 4023–4032.

PHILIP, R. & EPSTEIN, L. (1986). Tumour necrosis factor as immunomodulator and mediator of monocyte cytotoxicity induced by itself, γ-interferon and interleukin-1. *Nature*, **323**, 86–89.

RUGGIERO, U. & BAGLIONI, C. (1987). Synergistic anti proliferative activity of interleukin 1 and tumour necrosis factor. *J. Immunol.*, **138**, 661–663.

SATO, K., FUJI, Y., ONO, M., NOMURA, H. & SHIZUME, K. (1987). Production of interleukin 1 alpha-life factor and colony-stimulating factor by a squamous cell carcinoma of the thyroid (T3M-5) derived from a patient with hypercalcemia and leukocytosis. *Cancer Res.*, **47**, 6474–6480.

SHAU, H. (1988). Cytostatic and tumouricidal activities of tumour necrosis factor-treated neurtrophils. *Immunol. Lett.*, **17**, 47–51.

TAKEYAMA, H., WAKAMIYA, N., O'HARA, C., ARTHUR, K., NILOFF, J., KUFE, D., SAKARAI, K. & SPRIGGS, D. (1991). Tumour necrosis factor expression by human ovarian carcinoma *in vivo*. *Cancer Res.*, **51**, 4476–4480.

TSAI, S. & GAFFNEY, E.V. (1986). Inhibition of cell proliferation by interleukin 1 derived from monocytic leukemia cells. *Cancer Res.*, **46**, 1471–1477.

WARD, B.G., WALLACE, K., SHEPHERD, J.H. & BALKWILL, F.R. (1987). Intraperitoneal xenografts of human epithelial ovarian cancer in nude mice. *Cancer Res.*, **47**, 2662–2667.